# [^99m^Tc]Tilmanocept Accurately Detects Sentinel Lymph Nodes and Predicts Node Pathology Status in Patients with Oral Squamous Cell Carcinoma of the Head and Neck: Results of a Phase III Multi-institutional Trial

**DOI:** 10.1245/s10434-015-4382-x

**Published:** 2015-02-11

**Authors:** Amit Agrawal, Francisco J. Civantos, Kevin T. Brumund, Douglas B. Chepeha, Nathan C. Hall, William R. Carroll, Russell B. Smith, Robert P. Zitsch, Walter T. Lee, Yelizaveta Shnayder, David M. Cognetti, Karen T. Pitman, Dennis W. King, Lori A. Christman, Stephen Y. Lai

**Affiliations:** 1 Department of Otolaryngology—Head and Neck Surgery, Arthur G. James Cancer Hospital and Richard J. Solove Research Institute, The Ohio State University Wexner Medical Center, Columbus, OH USA; 2 Department of Otolaryngology, University of Miami Hospital and Clinics/Sylvester Comprehensive Cancer Center, Miami, FL USA; 3 Division of Head and Neck Surgery, Department of Surgery, Moores UCSD Cancer Center and Veteran Affairs San Diego Medical Center, San Diego, CA USA; 4 Department of Otolaryngology, University of Toronto, Toronto, ON Canada; 5 Division of Nuclear Medicine, Department of Radiology, The Ohio State University Wexner Medical Center, Columbus, OH USA; 6 Division of Otolaryngology—Head and Neck Surgery, Department of Surgery, University of Alabama at Birmingham, Birmingham, AL USA; 7 Department of Otolaryngology—Head and Neck Surgery, University of Nebraska Medical Center, Omaha, NE USA; 8 Department of Otolaryngology—Head and Neck Surgery, University of Missouri, Columbia, MO USA; 9 Division of Otolaryngology—Head and Neck Surgery, Department of Surgery, Duke University Medical Center, Durham, NC USA; 10 Department of Otolaryngology—Head and Neck Surgery, University of Kansas Medical Center, Kansas City, KS USA; 11 Department of Otolaryngology—Head and Neck Surgery, Thomas Jefferson University, Philadelphia, PA USA; 12 Banner MD Anderson Cancer Specialists, Gilbert, AZ USA; 13 STATKING Clinical Services, Fairfield, OH USA; 14 Department of Head and Neck Surgery, The University of Texas MD Anderson Cancer Center, Houston, TX USA

## Abstract

**Background:**

[^99m^Tc]Tilmanocept, a novel CD206 receptor-targeted radiopharmaceutical, was evaluated in an open-label, phase III trial to determine the false negative rate (FNR) of sentinel lymph node biopsy (SLNB) relative to the pathologic nodal status in patients with intraoral or cutaneous head and neck squamous cell carcinoma (HNSCC) undergoing tumor resection, SLNB, and planned elective neck dissection (END). Negative predictive value (NPV), overall accuracy of SLNB, and the impact of radiopharmaceutical injection timing relative to surgery were assessed.

**Methods and Findings:**

This multicenter, non-randomized, single-arm trial (ClinicalTrials.gov identifier NCT00911326) enrolled 101 patients with T1–T4, N0, and M0 HNSCC. Patients received 50 µg [^99m^Tc]tilmanocept radiolabeled with either 0.5 mCi (same day) or 2.0 mCi (next day), followed by lymphoscintigraphy, SLNB, and END. All excised tissues were evaluated for tissue type and tumor presence. [^99m^Tc]Tilmanocept identified one or more SLNs in 81 of 83 patients (97.6 %). Of 39 patients identified with any tumor-positive nodes (SLN or non-SLN), one patient had a single tumor-positive non-SLN in whom all SLNs were tumor-negative, yielding an FNR of 2.56 %; NPV was 97.8 % and overall accuracy was 98.8 %. No significant differences were observed between same-day and next-day procedures.

**Conclusions:**

Use of receptor-targeted [^99m^Tc]tilmanocept for lymphatic mapping allows for a high rate of SLN identification in patients with intraoral and cutaneous HNSCC. SLNB employing [^99m^Tc]tilmanocept accurately predicts the pathologic nodal status of intraoral HNSCC patients with low FNR, high NPV, and high overall accuracy. The use of [^99m^Tc]tilmanocept for SLNB in select patients may be appropriate and may obviate the need to perform more extensive procedures such as END.

Head and neck squamous cell carcinoma (HNSCC) of both mucosal and cutaneous origin carries variable propensity to metastasize to regional cervical nodes. The presence of nodal metastases is the most important negative prognostic factor for long-term survival.^1^–^3^ Thus, accurate identification and treatment of lymphatic metastases is important for this patient population.

As current methods, including physical examination and radiologic imaging, lack sufficient sensitivity and specificity,^4^,^5^ elective neck dissection (END) has been the gold standard for assessing the presence or absence of lymphatic disease in patients without overt clinical or radiographic nodal metastases (cN0) undergoing surgical management of HNSCC.^6^ However, END is associated with significant potential morbidity, including pain, contour changes, shoulder dysfunction, and lip paresis, as well as negative impact upon quality of life.^7^–^9^ Furthermore, it may be argued that END is unnecessary in a large proportion of patients; for example, 70–80 % of patients initially presenting with early-stage oral cavity carcinoma (T1 or T2, cN0) ultimately prove to be free of lymphatic metastases.^8^,^10^–^12^

Sentinel lymph node biopsy (SLNB) has been advocated as a less invasive means of achieving accurate diagnostic assessment of regional metastatic tumor potential while reducing morbidity compared with more extensive procedures.^9^

Several studies have examined SLNB in HNSCC using radiolabeled colloid.^13^–^18^ Despite excellent negative predictive values (NPV), the false negative rate (FNR) of SLNB for HNSCC (i.e. percentage of cases with overall positive END, SLN pathology-negative) appears variable and reached nearly 10 % in the two largest multicenter series.^14^,^18^ Characteristics of radiolabeled colloid, including its particulate nature and lack of specific binding, may in part contribute to observed FNR when used for SLNB in HNSCC.

[^99m^Tc]Tilmanocept, approved by the US FDA and recently granted marketing authorization by the European Medicine Agency’s Committee for Medicinal Products for Human Use for breast cancer, melanoma, and oral HNSCC SLN detection, is a novel, receptor-targeted, non-particulate radiopharmaceutical that consists of multiple diethylenetriaminepentaacetic acid (DTPA) molecules for ^99m^Tc chelation and mannose moieties for CD206 receptor binding tethered to a dextran scaffold. The small molecular size (7 nm diameter) of tilmanocept and its specific targeting to CD206 mannose-binding receptors located on reticuloendothelial cells within lymph nodes permit rapid injection site clearance and avid, stable binding within target nodes.^19^

This article describes the results of an open-label, FDA-designated, phase III trial to assess the accuracy of [^99m^Tc]tilmanocept used in conjunction with lymphoscintigraphy and SLNB to detect SLNs, as well as predict pathologic nodal status (i.e. presence vs. absence of metastatic disease) in patients with oral or cutaneous HNSCC undergoing SLNB and END.

## Methods

### Participants and Institutional Review/Consent

Eligibility criteria included T1–T4a, cN0, and M0 HNSCC located in the oral cavity or cutaneous head and neck region. Clinical nodal staging was confirmed by negative results from contrast-enhanced computed tomography (CT) scan, gadolinium-enhanced magnetic resonance imaging (MRI), or neck ultrasound. Patients with a history of neck dissection, gross injury to the neck, or radiotherapy to the neck or receiving systemic cytotoxic therapy were excluded from the trial.

Subject enrollment occurred across 13 centers. The protocol and informed consent were approved by the Institutional Review Boards of each center, and the study met all applicable regulatory and ethical requirements.

### Procedures

#### Radiopharmaceutical Injection and Lymphoscintigraphy

Patients received 50 μg of [^99m^Tc]tilmanocept radiolabeled with either 0.5 mCi (for surgeries on the same day as injection) or 2.0 mCi (for surgeries the day after injection). Timing of injection (i.e. day of surgery vs. day before surgery) was at the surgeon’s discretion, except in patients with floor-of-mouth tumors. In such patients, day-before-surgery injection was required to allow for significantly reduced shine-through, whereby radioactivity at the primary site may obscure relevant SLNs. Following injection, all patients underwent preoperative lymphoscintigraphy imaging per institutional protocol, which involved planar imaging (±dynamic) and/or fused single-photon emission computed tomography/CT (SPECT/CT).

#### Surgery/Sentinel Lymph Node Biopsy/Elective Neck Dissection

Surgery was required either within 1–15 h (same day) or 15–30 h (next day) following injection. At surgery, excision of the primary tumor was performed prior to SLNB/END. Using a handheld gamma detector, the surgeon conducted an initial survey of the entire cervical lymph node basin at risk to identify the areas of increased radioactivity. An SLN was defined as a lymph node with a mean in vivo count >3 square roots of the mean normal tissue background count (i.e. three standard deviations) added to the mean normal tissue background count (‘3*σ* rule’) asserting 99.7 % certainty of the SLN signal. As each SLN was identified and dissected, radioactivity counts were recorded in vivo and ex vivo. SLNB was considered complete when no further hot nodes were detected. Following SLNB, END was then performed. Bilateral ENDs were performed when the primary lesion involved the midline, tumors <1 cm from midline with evidence of contralateral drainage on lymphoscintigraphy, or per surgical discretion.

#### Histopathology Assessment of Lymph Nodes

All excised nodes (both SLNs and non-SLNs) underwent local routine histopathologic evaluation using hematoxylin and eosin (H&E) staining. After fixation, all SLNs were sectioned every 2 mm in transverse fashion along the longest axis and embedded into cassettes for sectioning, thus providing sections every 2–3 mm, producing at least three levels through the node for assessment. Additional staining was permitted locally based on institutional standards. All negative SLNs were sent to the study’s central pathology laboratory for additional immunohistochemical staining for pancytokeratin markers (e.g. AE1/AE3, CK8/18, MNF 116, etc.). All locally positive SLNs had two unstained slides sent to the central laboratory for confirmation of pathology positivity.

### Statistical Analyses

The primary endpoint was the FNR associated with assessment of [^99m^Tc]tilmanocept-identified SLNs relative to the overall pathologic nodal status as determined by assessment of both SLNs and non-SLNs from the END. The FNR is the ratio of false negatives to the sum of true positives plus false negatives. The overall FNR point estimate was the observed rate and was made on a per-patient basis relative to all patients with pathology-positive nodes. The statistical hypotheses *H*_0_: FNR ≥0.14 versus *H*_a_: FNR < 0.14, selected from an assessment of peer-reviewed publications of several prior studies examining SLNB in HNSCC, were tested using a one-sided significance level of 0.02486 such that if the upper limit of the 95.03 % confidence interval (CI) for the FNR was <0.14, the null hypothesis was rejected in favor of the alternative hypothesis. Exact binomial CIs were used.

Secondary patient-level measures of efficacy were NPV, overall accuracy of [^99m^Tc]tilmanocept, and rate of SLN detection by [^99m^Tc]tilmanocept. Point estimates for secondary endpoints were the observed rate; 95 % exact binomial CIs were calculated.

The intent-to-treat (ITT) population, consisting of all patients injected with [^99m^Tc]tilmanocept who underwent surgery and had at least one lymph node (SLN or non-SLN) with known pathology status, was used for all efficacy analyses.

## Results

### Demographics and Staging

Between June 2009 and November 2012, a total of 101 patients were enrolled. Of these, 16 patients withdrew from the study prior to drug administration or surgery—12 patients withdrew consent and four withdrew for other reasons. The remaining 85 patients were injected with [^99m^Tc]tilmanocept. The majority of patients had oral tumors (92.9 %) and either T1 or T2 (84.7 %) clinical staging (Table 1).

**Table 1:** Patient characteristics: ECOG status, tumor staging, and tumor location

Characteristic	No. of patients (%)		
	Cutaneous (*n* = 6)	Intraoral (*n* = 79)	Overall (*n* = 85)
Preoperative clinical T staging			
T1	0	26 (32.9)	26 (30.6)
T2	6 (100)	40 (50.6)	46 (54.1)
T3	0	7 (8.9)	7 (8.2)
T4	0	6 (7.6)	6 (7.1)
Preoperative clinical N staging			
N0	6 (100)	79 (100)	85 (100)
Preoperative clinical M staging			
M0	6 (100)	78 (98.7)	84 (98.8)
MX	0	1 (1.3)	1 (1.2)
ECOG performance status			
0	5 (83.3)	53 (67.1)	58 (68.2)
1	1 (16.7)	21 (26.6)	22 (25.9)
2	0	5 (6.3)	5 (5.9)

Data represent the [^99m^Tc]tilmanocept-injected population (*N* = 85)

*ECOG* Eastern Cooperative Oncology Group

### Imaging

**Fig. 1:**
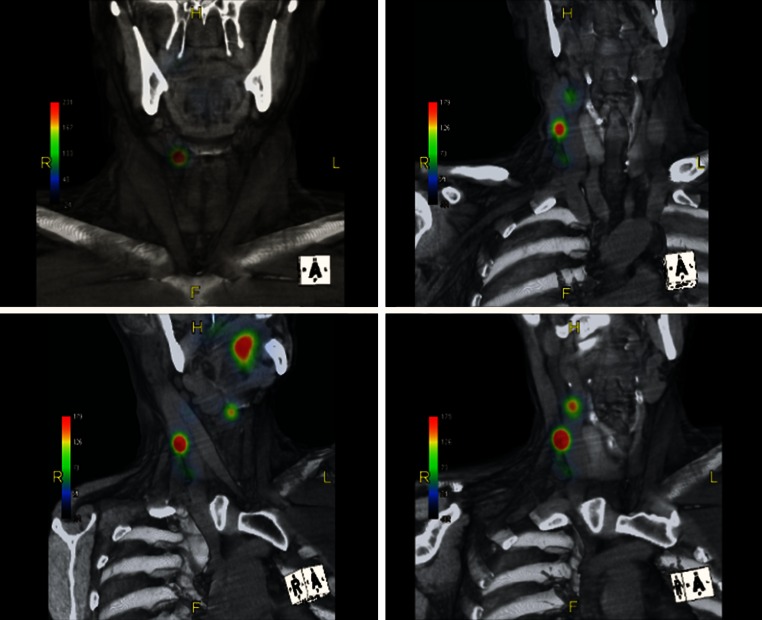
SPECT/CT three-dimensional fused reconstruction cross-sectional images of a typical patient with floor-of-mouth tumor (duration of SPECT/CT acquisition was 3–21 min post-injection of [^99m^Tc]tilmanocept. The *cube* in the lower right corner indicates the perspective of the image. *SPECT* single-photon emission computed tomography, *CT* computed tomography, *R* right, *L* left, *H* head, *F* feet, *A* anterior, *P* posterior

The preoperative SPECT/CT three-dimensional fused reconstruction cross-sectional images of a typical patient (image acquisition duration was 3–21 min) of [^99m^Tc]tilmanocept are shown in Fig. 1. SPECT/CT imaging revealed four SLNs in this patient by 21 min post-injection of [^99m^Tc]tilmanocept.

### Efficacy Measures

Of 85 patients injected with [^99m^Tc]tilmanocept, two patients did not undergo SLNB and END due to non-drug-related adverse events. Of note, there were no drug-related serious adverse events and no deaths on study. As such, 83 patients (78 intraoral and 5 cutaneous) injected with [^99m^Tc]tilmanocept underwent SLNB/END and comprised the ITT population for efficacy analyses.

At least one SLN was identified in 81 of the 83 ITT patients yielding an SLN detection rate of 97.6 %. Table 2 shows lymph node statistics by pathology and node type, as well as statistics according to whether SLN pathology was positive or negative per subject. Among the 83 ITT patients, a mean of 3.9 SLNs (median 4) were removed per patient (range 0–11 nodes). Of the non-SLNs obtained via END (i.e. following SLNB), a mean of 34.0 non-SLNs were removed per patient (range 0–82 nodes).

**Table 2:** Summary statistics for excised lymph nodes by pathology and per patient

Node type	Pathology status	Nodes per patient			
		Mean	95 % CI	Median	Range (min–max)
SLN (*n* = 323)	Overall	3.9	3.42–4.37	4	0–11
	Positive (*n* = 67)	0.8			
	Negative (*n* = 255)	3.1			
Non-SLN (*n* = 2,823)	Overall	34.0	30.02–38.01	30	0–82
	Positive (*n* = 21)	0.3			
	Negative (*n* = 2,802)	33.8			

Data represent the intent-to-treat population (*N* = 83)

*min* minimum, *max* maximum, *CI* confidence interval, *SLN* sentinel lymph node

In those subjects in whom one or more SLNs were pathology-positive for tumor, a mean of 4.5 SLNs (median 4.0) were removed per subject (range 2–11 nodes). In these same subjects, a mean of 32.5 non-SLNs (median 28.0) were removed via END (range 7–78 nodes).

Table 3 details SLN pathology status and overall nodal pathology status per subject, as well as efficacy metrics. Of the ITT patients, 39 (47.0 %), which were all intraoral patients, had at least one pathology-confirmed tumor-positive lymph node (SLN or non-SLN)—31 were staged T1–T2, and eight were staged T3–T4. The proportion of subjects identified with nodal tumor involvement was 44.3 % amongst patients with T1–T2 disease and 61.5 % amongst patients with T3–T4 disease. One patient (buccal mucosa tumor stage T2) in whom all SLNs identified by [^99m^Tc]tilmanocept were negative for tumor, had one tumor-positive node (non-SLN) which was not detected via SLNB using [^99m^Tc]tilmanocept (‘false negative’). The overall FNR was 2.56 %, with a 95.03 % CI of 0.06–13.49; thus, the prospectively established null hypothesis was rejected in favor of the alternative hypothesis (*p* = 0.0205). To the extent that all cutaneous tumor patients would be excluded from the FNR analysis, the FNR remains unchanged. Thirty-eight patients had at least one SLN that was tumor positive (‘true positives’). The FNR for the T1–T2 patients was 3.23 %, and 0 % for the T3–T4 patients. Forty-four of the patients in whom all SLNs were negative for tumor, as confirmed by the central laboratory, or in whom no SLNs were detected, also had all non-SLNs negative for tumor (both conditions included as ‘true negatives’). These data yielded an NPV of 97.8 % (Table 3). For the ITT population, overall accuracy of SLN identified via [^99m^Tc]tilmanocept in correctly determining the nodal pathology status of the neck was 98.8 %.

**Table 3:** Classification of patients according to pathology status of [^99m^Tc]tilmanocept-identified SLNs, overall pathology nodal status, and calculated efficacy performance metrics

	Overall nodal pathology status (SLN and non-SLN), by patient	
	Positive (with one or more nodes)	Negative
Pathology status of SLN, by patient		
Positive (one or more nodes)	38 (true positive)	–
Negative (or no SLNs identified)	1 (false negative)	44 (true negative)

Performance metrics	Rate	95 % exact binomial CI^a^
False negative rate	0.0256	0.0006–0.1349
Negative predictive value	0.9778	0.8823– 0.9994
Overall accuracy	0.9880	0.9347– 0.9997

Data represent the intent-to-treat population (*N* = 83)

*CI* confidence interval, *SLN* sentinel lymph node

^a^The CI for the false negative rate is 95.03 %

Pathology-positive and false-negative patients by tumor location and timing of surgery are shown in Table 4. No differences in FNR were observed between individual tumor subsites or between same-day and next-day procedures.

**Table 4:** Summary of patients by tumor location and time of surgery

Variable	Total ITT patients	Patients with SLNs detected	All pathology-positive patients	False negative patients
Tumor location				
Buccal mucosa	8	8	4	1
Cutaneous	5	4	0	0
Floor of mouth	20	20	12	0
Lower alveolar ridge	3	3	2	0
Mucosal lip	1	1	0	0
Oral tongue	42	42	21	0
Retromolar gingiva	4	3	0	0
Time of surgery^a^				
Same day	40	40	22	1
Next day	42	40	16	0

Data represent the ITT population (*N* = 83)

*ITT* intent-to-treat, *SLNs* sentinel lymph nodes

^a^Time of surgery was missing for one patient and could therefore not be included in the time-of-surgery analyses

### Data and Safety Monitoring

The current study was overseen by an independent Data and Safety Monitoring Committee (DSMC). The study was prospectively structured to include an interim analysis at 33.3 % (*N* ≥ 38) of the targeted accrual cohort (*N* ≥ 114) of node pathology-positive subjects. The trial was terminated early based on an interim review by the DSMC due to positive efficacy outcome. The DSMC noted that as the study achieved its primary efficacy endpoint, the added risk of END may not be justified in those situations where SLN assessment determined node-negative status.

## Discussion

Although routine in the management of breast cancer and melanoma, the use of SLNB procedures for HNSCC continues to evolve. Two large, multicenter, prospective trials to date have described SLNB for HNSCC using radiolabeled colloid with or without blue dye. A prospective trial at six centers in Europe followed 134 patients with T1–T2 N0 tumors of the oral cavity or oropharynx who either underwent SLNB alone or in SLNB in combination with END. In this trial, the FNR of SLNB after long-term follow-up was 9 %.^18^,^20^ A prospective multi-institutional cooperative group trial (Z-0360) carried out in the US and sponsored by the American College of Surgeons Oncology Group (ACOSOG), involving 25 institutions over a 3-year period, assessed 140 patients with T1 and T2 oral cavity carcinoma. In this group, the NPV of SLNB was 96 %, with an observed FNR of 9.8 %.^14^

Despite the difference between studies in the number of subjects in the ITT population (ACOSOG Z-0360 study: 140 subjects; NEO3-06 study: 83 subjects), there was a similar number of node pathology-positive subjects (ACOSOG Z-0360: 41 subjects; NEO3-06: 39 subjects), which serves as the basis for the comparison of these studies.^14^,^21^ In the current study, the FNR of [^99m^Tc]tilmanocept (2.56 %) was statistically significantly lower than the upper limit of the FNR of [^99m^Tc]sulfur colloid noted in the ACOSOG Z-0360 study (observed FNR of 9.8 %, 95 % CI 2.7–23.1; *p* = 0.0005). The accuracy of [^99m^Tc]tilmanocept was also statistically significantly greater than the lower limit of the accuracy of [^99m^Tc]sulfur colloid as used in the Z-0360 study (*p* = 0.0151).^21^

Several contributing factors have been noted regarding the observed variable FNR for SLNB using radiolabeled colloid for HNSCC, including tumor location (floor-of-mouth tumors with higher FNR) and larger tumors (i.e. T2 vs. T1).^14^,^18^ Due to its particulate nature and non-standardized preparation, radiolabeled colloids (100–1,000 nm particle diameter) are retained for prolonged periods within the injection site, which in turn contributes to the phenomenon of shine-through effect.^22^ This is particularly problematic for floor-of-mouth tumors which, in previous studies, have been associated with significantly lower rates of SLN identification (88 %) and higher FNRs (20 %) compared with other oral sites.^18^,^20^ In comparison, the current trial included 20 patients with floor-of-mouth tumors, of whom [^99m^Tc]tilmanocept identified at least one SLN in all patients (100 %). Twelve of these patients were identified with metastatic nodal disease and, in all 12, at least one SLN was identified with metastatic disease. As such, the observed NPV and overall accuracy of SLNB using [^99m^Tc]tilmanocept in this group of patients was 100 %.

Criticism of the current study could focus on the inclusion of patients with larger tumors (higher expected nodal metastatic rate), as well as those with cutaneous HNSCC (lower expected nodal metastatic rate). Patients with larger tumors (T3, T4) comprised a relatively small group overall (13 patients, 15 %), but these patients were included as all patients were planned to undergo standard-of-care END. Given the high rate of occult nodal disease observed in these patients (8 of 13 patients, 61.5 %), one might reasonably forgo SLNB in favor of planned (i.e. therapeutic) END; however, in this study, the FNR for this subpopulation was 0 %. While the use of SLNB alone in patients with larger tumors is certainly controversial, lymphatic mapping procedures in such patients undergoing planned END (i.e. ‘SLN-assisted END’) might identify additional neck regions at risk, including the contralateral neck, not routinely encompassed during END alone. As such, the concept of SLNB procedures in this population may warrant further investigation. Patients with cutaneous HNSCC were a relatively small cohort (five patients, 6 %). None were found to have nodal disease following SLNB and END. The lack of observed nodal metastases in these patients limits the assessment of predictive utility of [^99m^Tc]tilmanocept for SLNB (i.e. FNR, NPV) as related to cutaneous HNSCC, and also indicates the need for further study.

Of note, the specificity of tilmanocept for lymphatic tissues assessed via in vivo imaging and in vitro analysis of its receptor binding properties suggest that tilmanocept does not move downstream to distal lymph nodes, permitting high confidence that a hot node found during next-day procedures is in fact an SLN.^19^ The present study supports that the SLN detection rate and FNR for nodal metastases were not significantly affected by the day of surgery relative to timing of [^99m^Tc]tilmanocept injection. This attribute portends that the use of [^99m^Tc]tilmanocept provides substantial leeway and scheduling flexibility with regard to time of injection and subsequent lymphoscintigraphy and SLNB procedures (i.e. next-day surgery) without compromising the reliability of results.

## Conclusions

The current trial supports the use of [^99m^Tc]tilmanocept in the setting of SLNB for HNSCC with a high rate of SLN identification. When used in conjunction with serial sectioning and immunohistochemistry, SLNB with [^99m^Tc]tilmanocept accurately predicts the nodal pathology status of the neck in patients with oral HNSCC with low FNR, high NPV, and high overall accuracy. Given these results, the use of [^99m^Tc]tilmanocept in this setting may help surgeons avoid the need to perform more extensive procedures, including END.

## Appendix: Investigators and Enrolling Institutions

Amit Agrawal, MD

Department of Otolaryngology—Head and Neck Surgery, Arthur G. James Cancer Hospital and Richard J. Solove Research Institute, The Ohio State University Wexner Medical Center, Columbus, OH, USA

Stephen Y. Lai, MD, PhD

Department of Head and Neck Surgery, The University of Texas MD Anderson Cancer Center, Houston, TX, USA

Kevin T. Brumund, MD

Department of Surgery, Division of Head and Neck Surgery, Moores UCSD Cancer Center and Veteran Affairs San Diego Medical Center, San Diego, CA, USA

Francisco J. Civantos, MD

Department of Otolaryngology, University of Miami Hospital and Clinics/Sylvester Comprehensive Cancer Center, Miami, FL, USA

Douglas B. Chepeha, MD

Department of Otolaryngology, University of Michigan, Ann Arbor, MI, USA

William R. Carroll, MD

Department of Surgery, Division of Otolaryngology—Head and Neck Surgery, University of Alabama at Birmingham, Birmingham, AL, USA

Russell B. Smith, MD

Department of Otolaryngology—Head and Neck Surgery, University of Nebraska Medical Center, Omaha, NE, USA

Robert P. Zitsch, MD

Department of Otolaryngology—Head and Neck Surgery, University of Missouri, Columbia, MO, USA

Walter T. Lee, MD

Department of Surgery, Division of Otolaryngology—Head and Neck Surgery, Duke University Medical Center, Durham, NC, USA

Yelizaveta Shnayder, MD

Department of Otolaryngology—Head and Neck Surgery, University of Kansas Medical Center, Kansas City, KS, USA

David M. Cognetti, MD

Department of Otolaryngology—Head and Neck Surgery, Thomas Jefferson University, Philadelphia, PA, USA

Karen T. Pitman, MD

Department of Otolaryngology, University of Mississippi Medical Center, Jackson, MS, USA
